# Tricarboxylic Acid Metabolite Imbalance in Rats with Acute Thioacetamide-Induced Hepatic Encephalopathy Indicates Incomplete Recovery

**DOI:** 10.3390/ijms24021384

**Published:** 2023-01-10

**Authors:** Yevgeniya I. Shurubor, Alexander E. Rogozhin, Elena P. Isakova, Yulia I. Deryabina, Boris F. Krasnikov

**Affiliations:** 1 Centre for Strategic Planning and Management of Medical and Biological Health Risks, Federal Medical Biological Agency of The Russian Federation, Moscow 119121, Russia; 2 Valiev Institute of Physics and Technology of the Russian Academy of Sciences, Moscow 117218, Russia; 3 Bach Institute of Biochemistry, Research Center of Biotechnology of the Russian Academy of Sciences, Moscow 119071, Russia

**Keywords:** hepatic encephalopathy, thioacetamide, HPLC, metabolites, tricarboxylic acid cycle metabolites, α-ketoglutaramate

## Abstract

Exposure to the toxin thioacetamide (TAA) causes acute hepatic encephalopathy (HE), changes in the functioning of systemic organs, and an imbalance in a number of energy metabolites. The deferred effects after acute HE development are poorly understood. The study considers the balance of the tricarboxylic acid (TCA) cycle metabolites in the blood plasma, liver, kidneys, and brain tissues of rats in the post-rehabilitation period. The samples of the control (n = 3) and TAA-induced groups of rats (n = 13) were collected six days after the administration of a single intraperitoneal TAA injection at doses of 200, 400, and 600 mg/kg. Despite the complete physiological recovery of rats by this date, a residual imbalance of metabolites in all the vital organs was noted. The results obtained showed a trend of stabilizing processes in the main organs of the animals and permit the use of these data both for prognostic purposes and the choice of potential therapeutic agents.

## 1. Introduction

Hepatic encephalopathy (HE) is a potentially reversible neurodegenerative pathology resulting from either acute or chronically progressive liver failure [1].

HE develops due to poisoning the body with toxic substances or ammonia that are not excreted in time by the liver because of its functional impairment. Liver malfunction caused by toxic substances also affects brain functioning. The resulting changes are based on mitochondrial dysfunction, namely: impaired permeability of mitochondrial membranes, oxidative stress, disruptions of the tricarboxylic acid (TCA) cycle, inhibition of the activity of α-ketoglutarate dehydrogenase (α-KGDH), pyruvate dehydrogenase (PDH), and a number of other important enzymes [2,3,4].

The effect of HE on the organism is better considered as an integrated influence that damages vital organs (liver, kidneys, and brain) and affects circulating blood, which takes part in cleansing the body. The liver plays an important role in converting ammonia into urea and removing toxins from the body with urine. Deterioration in liver functioning due to its overload with harmful substances leads to the accumulation of toxins in the kidney, degradation, and failure of the organ [2,5]. An important resource for cleaning the body from toxins is the circulatory blood flow. Organ dysfunction, reduced blood flow, and poor cerebral oxygen uptake lead to the accumulation of toxins in brain tissues, which is often observed in patients with clinically advanced HE and/or cirrhosis of the liver [2].

To study HE, TAA models of rats have been used, which differ both in the doses of the applied toxin (200–900 mg/kg) and in the frequency of its exposure (single/multiple) [6,7,8]. It has been shown that the optimal dose of TAA for creating a model of acute HE in rats is a dose of 350 mg/kg [9].

Some other authors have shown that liver necrosis, which occurs with a single intraperitoneal administration of TAA at a dose of ~500 mg/kg, is replaced by its complete regeneration already three days after intoxication. Maximal death of rat liver cells is observed on the first day of TAA administration, and complete recovery of the hepatocyte population is observed on the third day after intoxication [10]. The aim of our study was to test the hypothesis that there is no statistically significant difference in the metabolic profile between the groups of rats after a single-dose administration of TAA followed by a six-day recovery period and rats that are not exposed to TAA.

The development of HE affects the production of a number of coenzymes and metabolites of TCA [11,12,13,14]. Previously, Cooper and Kuhara showed that the level of a potential biomarker-metabolite α-ketoglutaramate (α-KGM) in the cerebrospinal fluid of patients with liver diseases can be used to estimate the degree of development of hyperammonemia [15]. In this context, this is perhaps the only study in the field of hyperammonemia. Additional information on the content of α-KGM in blood plasma and tissues of human and/or animal organs is not yet available. Presumably, this is due to the existing difficulties in the determination of α-KGM in biological samples, as well as the commercial unavailability of α-KGM for the preparation of a standard solution. Previously, we have developed methods for simple and inexpensive determination of α-KGM in biological samples (based on the use of HPLC-UV), as well as laboratory synthesis of highly purified α-KGM [16,17].

Using the previously proposed methods for the determination of α-KGM and TCA metabolites in blood plasma and tissue samples obtained from rats [16,18], trends were established in the distribution of major energy metabolites depending on the dose of TAA in the post-recovery period of HE development.

## 2. Results

The physiological state of rats that received doses of TAA at 200, 400, and 600 mg/kg was different. Animals that received TAA at a dose of 200 mg/kg a day later looked healthy, while rats that received TAA at doses of 400–600 mg/kg were somewhat depressed. It should be noted that within each of the considered groups of animals, the level of biological (individual) variability of a number of metabolites in response to the action of the toxin turned out to be relatively high. Two rats from the group to which TAA was introduced at a dose of 600 mg/kg did not withstand the effects of the toxin and died within the next day. After 2–3 days, the remaining animals looked fully adapted of the toxin action. To assess the metabolic balance of rats that recovered after exposure to TAA, the levels of TCA metabolites and their associated compounds were measured: oxaloacetate (OA), citrate (Cit), isocitrate (Iso), α-ketoglutarate (α-KG), succinate (Suc), succinyl-coenzyme A (SCoA), malate (Mal), fumarate (Fum), pyruvate (Pyr), lactate (Lac), and α-KGM.

### 2.1. Rat Blood Plasma

**Figure 1:**
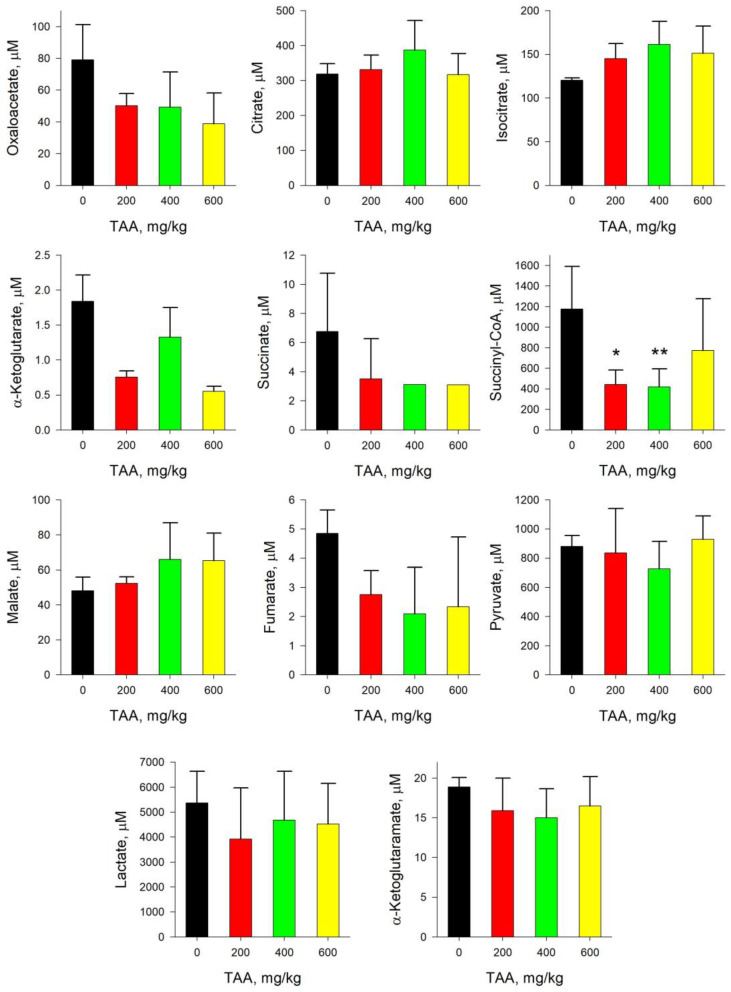
Concentration of TCA cycle and associated metabolites in the blood plasma of control and TAA-treated rats (AVG ± STDEV, µM). *p*-values by Tukey’s test: *—*p* < 0.1; **—*p* < 0.05; black stars mean a pairwise comparison to control (for details see Table and Figures in Supplement S1).

In the blood plasma of TAA-treated rats after the post-rehabilitation period, the concentration of most of the metabolites (OA, SCoA, Pyr, α-KG, α-KGM, Fum, Lac, Suc) was lower than the control values, while the concentration of the remaining metabolites (Cit, Iso, Mal) exceeded the control values (Figure 1). The most noticeable (≥2-fold) decrease in concentration with an increase in the dose of TAA relative to control values was noted in OA, SCoA, α-KG, Fum, Suc, and a relatively small decrease (10–20%) in Pyr, α-KGM, and Lac. The upward trends in metabolites such as Cit, Iso, and Mal were also small, no more than 10–30%.

**Figure 2:**
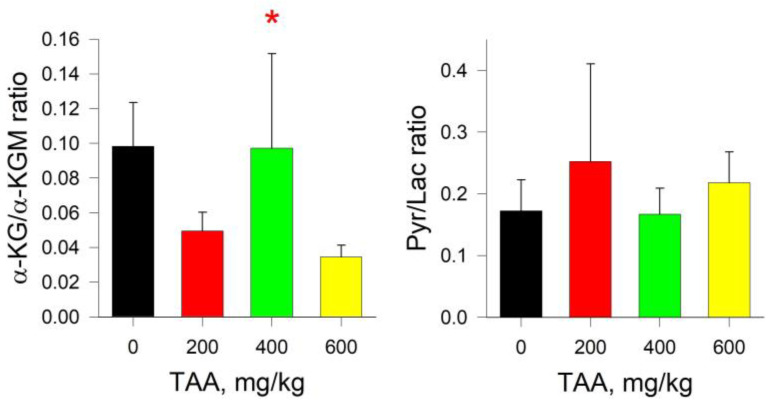
α-KG/α-KGM and Pyr/Lac ratios for blood plasma control and TAA-treated rats. *p*-values by Tukey’s test: *—*p* < 0.1; red stars mean a pairwise comparison to TAA600 (for details see Table and Figures in Supplement S1).

Of particular interest is the comparison of trends in the distribution of such reference metabolites as Pyr and Lac, α-KG and α-KGM. The concentrations of Pyr and Lac in the groups of TAA-induced rats decreased slightly relative to the control values, by ~10 and ~20%, respectively. The absolute concentration of α-KG (1.9 µM) in the blood plasma of the control group of rats was an order of magnitude lower than the concentration of α-KGM (18.9 µM). In the group of TAA-induced rats, the concentration of both of these metabolites was also below the control values (Figure 1), and the decrease in the level of α-KG was ~30–70%, and that of α-KGM was ~15–20%. In this regard, the ratio of α-KG/α-KGM in the control group of rats was ~0.1 (mean value), and in the groups of TAA-induced rats it varied in the range from 0.03 up to 0.1 (mean value), with lowest value at the highest concentration of TAA. The distribution of Pyr/Lac ratios in TAA-induced groups of rats did not vary much from that of control (~0.17) and were in the range of around 0.17–0.25 (mean value) (Figure 2).

### 2.2. Rat Liver

**Figure 3:**
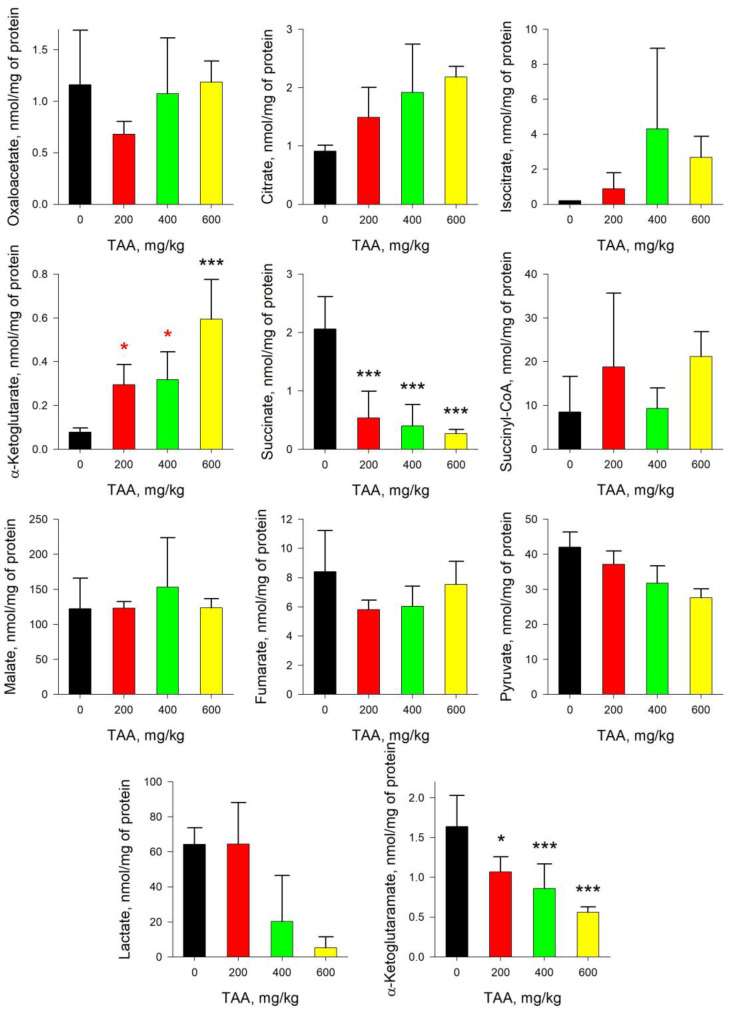
Concentration of TCA cycle and associated metabolites in the liver tissue of control and TAA-treated rats (AVG ± STDEV, nmol/mg of protein). *p*-values by Tukey’s test: *—*p* < 0.1; ***—*p* < 0.005; black stars mean a pairwise comparison to control, red mean a pairwise comparison to TAA600 (for details see Table and Figures in Supplement S1).

In the tissues of the liver of rats of the post-rehabilitation period, an imbalance in the concentrations of TCA and associated metabolites was also observed (Figure 3). Moreover, their disproportions in the liver of TAA-induced rats were more pronounced than in blood plasma. Thus, relative to control in mean values, there was an increase in the levels of Iso (≥10-fold), α-KG (≥6-fold), OA (≥2-fold), SCoA (≥2-fold), Cit (≥2-fold), and Mal (≥1.1-fold), and decreased levels of Lac (≥10-fold), α-KGM (≥3-fold), Pyr (≥2-fold), and Fum (≥1.1-fold).

**Figure 4:**
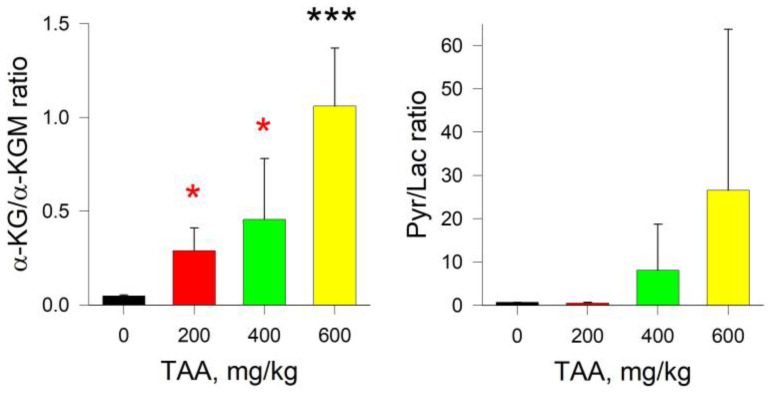
α-KG/α-KGM and Pyr/Lac ratios for liver tissue control and TAA-treated rats. *p*-values by Tukey’s test: *—*p* < 0.1; ***—*p* < 0.005; black stars mean a pairwise comparison to control, red—pairwise comparison to TAA600 (for details see Table and Figures in Supplement S1).

The absolute content of α-KG and α-KGM in the liver tissues of the control group of rats was 0.08 and 1.64 nmol/mg of protein, respectively, that is, it already differed by two orders of magnitude. Moreover, if in the blood plasma of rats exposed to TAA the concentration of α-KG decreased, then in the liver of the same groups of rats, the concentration of α-KG increased significantly (~8-fold) (Figure 1 and Figure 3). Curiously, an increase in the level of α-KG in the liver of TAA-induced rats was accompanied by a synchronous decrease in the level of α-KGM. In contrast to a slight decrease in the level of α-KGM in the blood plasma of TAA-induced rats, in the liver of animals this decrease was ~3-fold. The ratio of α-KG/α-KGM in liver tissues ranged from ~0.05 in the control group of rats to 1.06 (mean value) in the group of rats with a high dose of administration of TAA (600 mg/kg), that is, up to a ≥20-fold difference. The distribution of Pyr/Lac ratios in the liver tissues of rats that received various doses of TAA (as well as in blood plasma) varied in the range from ~0.5 in the control group of rats up to ~28 (mean value) in the group of rats that received high doses of TAA (600 mg/kg). Although the levels of both metabolites in the rat liver decreased with an increasing dose of TAA, the decrease in the level of Lac was almost eight times greater than the decrease in the level of Pyr (Figure 4).

### 2.3. Rat Kidney

In the kidney tissues of experimental rats, the imbalance in the content of TCA and associated metabolites was somewhat different from that in the liver (Figure 2 and Figure 3). The concentrations of Fum, Lac, Suc, Mal, and Iso in TAA-treated rats by mean values were ~1.5–5 times higher than those in the control, while the concentrations of OA, Pyr, SCoA, α-KG, and α-KGM were ~1.5–4 times lower. Trends in the behavior of metabolites such as Pyr, α-KGM, Iso, and Mal in the kidneys and liver of TAA-induced rats coincided, while those of SCoA, OA, α-KG, Cit, Fum, Lac, and Suc were multidirectional.

**Figure 5:**
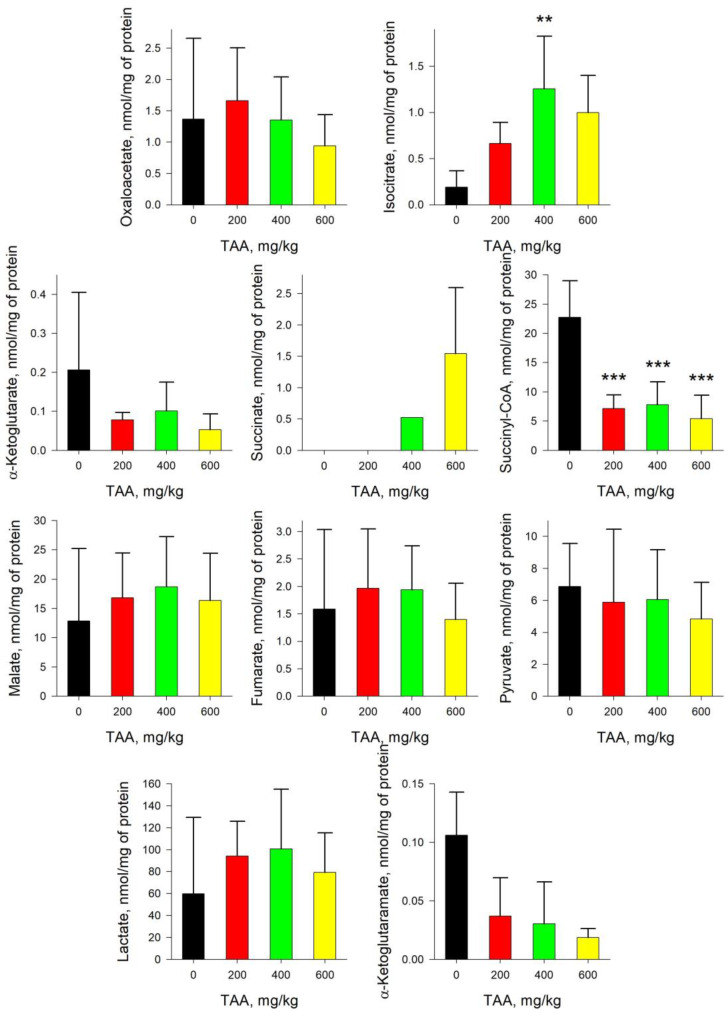
Concentration of TCA cycle and associated metabolites in the kidney tissue of control and TAA-treated rats (AVG ± STDEV, nmol/mg of protein). *p*-values by Tukey’s test: **—*p* < 0.05; ***—*p* < 0.005; black stars mean a pairwise comparison to control (for details see Table and Figures in Supplement S1).

Unlike both blood plasma and liver tissues, the amount of α-KG and α-KGM in the kidney tissues of the control and TAA-induced groups of rats differed slightly, only up to two times: 0.21 and 0.11 nmol/mg of protein, respectively (Figure 5). Noteworthy is the relatively low content of α-KGM in the tissues of the kidneys compared with that in the liver. As well as in blood plasma, the concentration of α-KG and α-KGM in the groups of TAA-induced rats was lower than the control values. However, this decrease was much more pronounced and was, respectively, ~4–6-fold.

Despite the simultaneous decrease in the levels of both metabolites in the kidney of TAA-induced rats, the change in the ratio of α-KG/α-KGM relative to the control level varied from around 2.5–3 times up to ~6-fold. 

**Figure 6:**
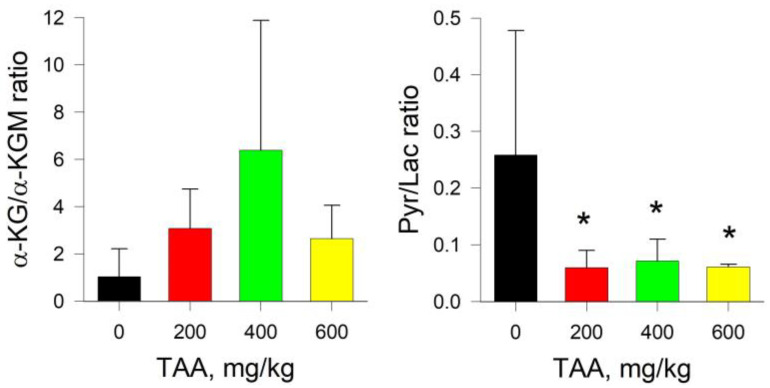
α-KG/α-KGM and Pyr/Lac ratios for kidney tissue of control and TAA-treated rats. *p*-values by Tukey’s test: *—*p* < 0.1; black stars mean a pairwise comparison to control (for details see Table and Figures in Supplement S1).

Pyr levels in the rat groups slightly decreased with increasing TAA doses, while Lac increased, so the Pyr/Lac ratios remained approximately equal to ~0.06 against control values around 4-fold as high as ~0.26 (mean value) (Figure 6). Thus, in the kidneys of rats, the trends in the ratios of α-KG/α-KGM, in contrast to those in blood plasma, were to some extent reversed.

### 2.4. Rat Brain

**Figure 7:**
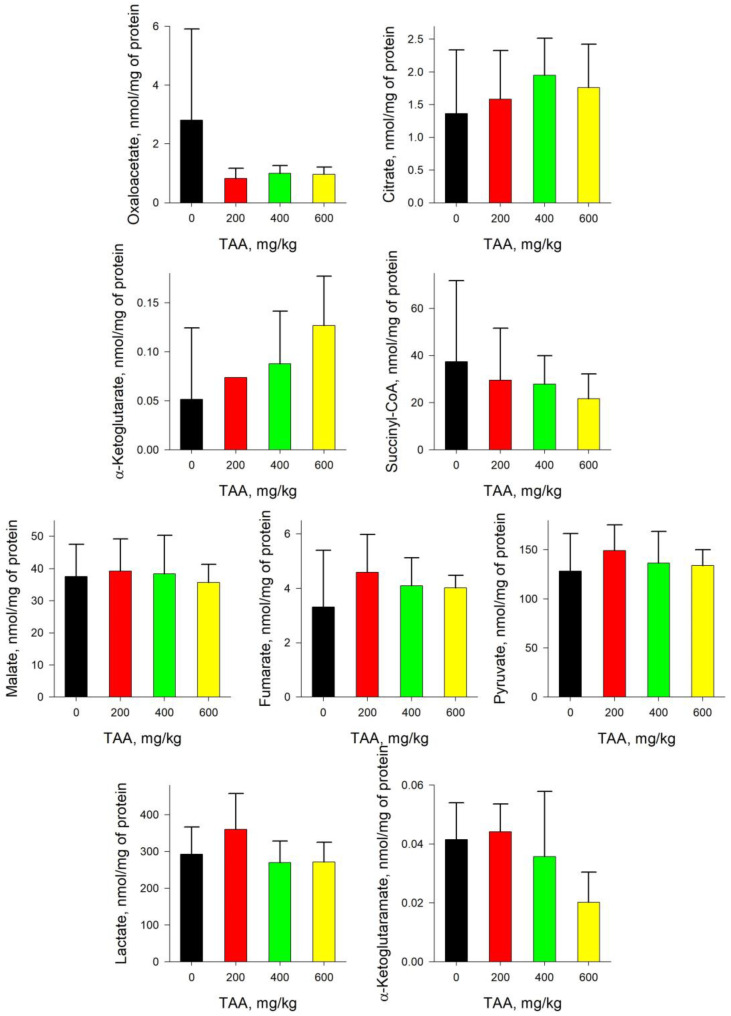
Concentration of TCA cycle and associated metabolites in the brain tissue of control and TAA-treated rats (AVG ± STDEV, nmol/mg of protein). According to results obtained by Tukey’s test, all data were not significantly different.

In the brain tissue of TAA-induced rats, compared to that of the control, there was a mean value increase in the concentrations of α-KG (≥2.5-fold), Cit (1.4-fold), Fum (1.4-fold), Lac (1.3-fold), and Pyr (1.2-fold), and a decrease in the concentrations α-KGM (≥2-fold), SCoA (1.4-fold), and OA (1.2-fold) (Figure 7). The absolute levels of α-KG and α-KGM in the brain tissues of the animals of the control group were quite similar and amounted to 0.05 and 0.04 nmol/mg of protein, respectively. A discrepancy in concentrations was noted in groups of rats that had previously undergone TAA intoxication. Trends in the distribution of α-KG and α-KGM in brain tissues of TAA-induced rats were similar to those in liver tissues (see Figure 3 and Figure 7). The increase in α-KG levels relative to control values was ~2-fold (0.05 and 0.12 nmol/mg of protein), and the decrease in α-KGM levels was also ~2-fold (0.02 and 0.04 nmol/mg of protein).

**Figure 8:**
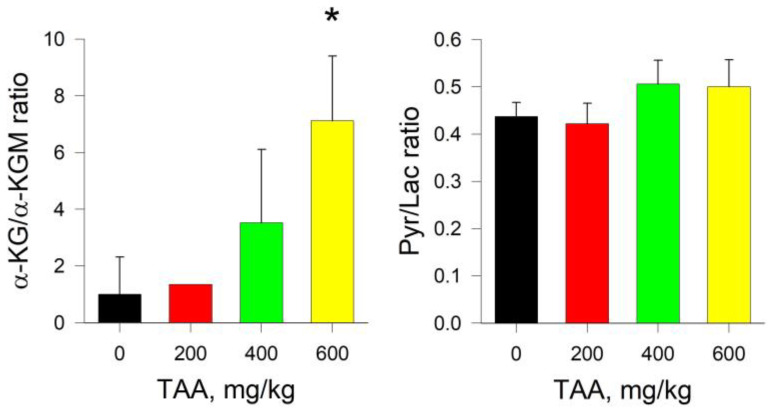
α-KG/α-KGM and Pyr/Lac ratios for brain tissue of control and TAA-treated rats. *p*-values by Tukey’s test: *—*p* < 0.1; black stars mean a pairwise comparison to control (for details see Table and Figures in Supplement S1).

The ratio of α-KG/α-KGM in the brain tissues of rats increased from the control group to the group with the maximal level of exposure to the toxin by about seven times (from around mean value 1 up to ~7, Figure 8). In contrast to the distribution of α-KG and α-KGM in the brain tissues of TAA-induced rats, Pyr and Lac levels changed very slightly; as a result, the Pyr/Lac ratio remained almost unchanged and was in the range of ~0.4–0.5.

## 3. Discussion

The metabolic consequences of the energy imbalance in the organs of rats that have recovered from acute HE are considered. It is assumed that an important role in the pathogenesis of HE is played by an increased level of ammonia in the blood and brain [19], which contributes to an increase in glycolysis, a decrease in the rate of TCA activity, and an increase in the consumption of α-KG for ammonia detoxification [20].

An increase in the rate of glycolysis with an excess of ammonia is associated with the activation of a number of enzymes, including pyruvate kinase [21]. It is assumed that an increase in the rate of glycolysis should increase the rate of TCA, but usually this does not happen, most likely due to the inhibition of PDH by ammonia, when Pyr formed as a result of glycolysis is predominantly converted to Lac [22,23]. A high level of Lac in the blood is often accompanied by cerebral edema and increased intracranial pressure [24]. At the same time, a decrease in the level of Pyr and an increase in the level of Lac in the blood and brain, expressed as a decrease in the Pyr/Lac ratio, is considered as one of the prognostic markers for the development of HE [25,26,27].

With the development of HE, part of the free ammonia in the blood in the brain tissues is also spent on the formation of glutamate; therefore, the content of α-KG, the substrate necessary for the formation of glutamate, also decreases [28]. In addition, ammonia inhibits α-KGDH in brain tissues [29]. Simultaneous decrease in α-KG reserves and inhibition of α-KGDH under conditions of acute HE can also slow down the work of TCA and affect the bioenergetics of the brain. Inhibition of isocitrate-, malate dehydrogenase, and a number of other enzymes can contribute to slowing down the work of TCA under the influence of excess ammonia [22,23].

The slowdown of the TCA cycle under the influence of ammonia contributes to a decrease in mitochondrial oxidative phosphorylation and reduces the reserves of ATP and other high-energy metabolites [30,31]. It is well documented in an experiment with the use of TAA in triplicates at a dose of 300 mg/kg of body weight, which resulted in a ≥30% decrease in ATP relative to baseline values [32].

In addition to changes in Lac levels, the level of α-KGM in the cerebrospinal fluid (CSF) of patients can act as a potential biomarker for the degree of development of HE. It was shown that the level of α-KGM in the CSF correlates well with the levels of ammonia and glutamine [15]. The mechanism of this process is not entirely clear, but there is an assumption that an increase in the level of α-KGM in the CSF in patients with HE may be partially due to an increase in the content of glutamine in the brain and α-ketoacid substrate for glutamine transaminases [15]. It was also noted that the level of α-KGM in the urine of patients can serve as a biomarker for some hyperammonemic diseases associated with a violation of the urea cycle, lysinuric protein intolerance, and Cit deficiency [33].

Our data indicate that the consequences of the development of acute HE under the influence of TAA persisted at the metabolic level of rats much longer than at the physiological level. Residual imbalance of TCA metabolites in plasma and tissues of rat organs (Figure 1, Figure 2, Figure 3, Figure 4, Figure 5, Figure 6, Figure 7 and Figure 8) was recognized even after the period of complete recovery of animals after the development of acute HE [10]. In general, higher doses of a toxicant cause more severe symptoms and last longer. In our study, the changes induced by TAA in relation to a number of metabolites were consistent with this concept. However, some metabolites did not quite fit into this scheme. It is likely that this may be due to, as we noted above, the high level of individual metabolite variability in animals in response to TAA exposure. This is especially indicative in the example we have considered below with α-KG and α-KGM.

It should be noted that the content of metabolites in blood plasma is not a direct indicator, but only a reflection of the direction of processes in various organs and tissues of experimental animals. The individual biological variability of TCA metabolites in the blood plasma of rats, both within one and between different groups, was quite high. This is not surprising, since the level of individual variability of metabolites in healthy people usually reaches ~50–70%, and the level of TCA metabolites in muscle tissues easily increases ~2–3 times even with short (5–10 min) physical activity [34,35].

At the same time, despite the presence of high individual biological variability of metabolites and a long period of recovery in rats after exposure to a toxin, the intergroup content of metabolites in the blood plasma of rats differed (Figure 1). Thus, by the mean values, the concentrations of Cit, Iso, Mal, and ACoA (according to [13]) were slightly (~10–30%) increased in the groups of TAA-induced rats. The concentration of most other metabolites (SCoA, OA, Pyr, Lac, α-KGM, α-KG, Fum, and Suc) was, on the contrary, decreased. The highest decrease (≥2 times) in the concentration in the group of TAA-induced rats was observed in OA, SCoA, α-KG, Fum, and Suc, and a slight decrease (10–20%) in Pyr, α-KGM, and Lac. 

As already noted, in hyperammonemic conditions and the development of HE, the Pyr/Lac ratio usually decreases due to a decrease in the level of Pyr and an increase in the level of Lac [25,26,27]. With regard to this criterion for assessing the degree of development of HE (Pyr/Lac), then in blood plasma, its special correlation with the severity of the previously transferred disease was not revealed. Thus, the content of Pyr gradually decreased in the groups with low and medium doses of TAA and slightly increased in the group with a high dose of TAA (600 mg/kg), which may be due to the biological variability of the metabolite (Figure 1). The level of Lac also decreased in all groups of TAA-induced rats relative to control values, but this was most noticeable in the group of rats with the minimal dose of administration of TAA (200 mg/kg). In this regard, the Pyr/Lac ratio was somewhat lower in the groups with low and high doses of TAA applied, and in the group of rats with an average dose of TAA it remained close to value of the control group (Figure 2).

Perhaps these small intergroup changes should not have been discussed had a similar trend not been found in another potential biomarker for the development of HE, the α-KG/α-KGM ratio (Figure 2). The intergroup distribution of α-KGM levels practically repeated the distribution pattern of Pyr. On the other hand, the distribution pattern of α-KG was close to that of Lac. It is possible that the analogies drawn in the synchronization of these trends are associated with the individual characteristics of animals. However, there is a possibility that this may be associated with the work of the corresponding blood enzymes and/or organs of rats, especially if we consider blood plasma as an indirect characteristic of the state of various organs.

In general, the functioning of TCA is maintained due to the balance of anaplerotic and cataplerotic reactions of a number of intermediate metabolites. The central process in it is the oxidation of ACoA to CO_2_ [35]. In cataplerosis, intermediate metabolites, leaving TCA, participate in various biosynthetic pathways and are converted into glucose, fatty acids, or non-essential amino acids. To replenish the declining metabolites and achieve a balance in the work of TCA, as a result of anaplerotic reactions, new intermediate metabolites are formed. An example is the formation of OA under the influence of the enzyme pyruvate carboxylase.

The quantitative change in the content of metabolites in the liver of rats is more pronounced than that in blood plasma, since the liver is saturated with specific enzymes and is the first to take a hit as a result of the development of acute HE (Figure 3). After the rehabilitation period, in the liver of TAA-induced rats, the levels of Mal, Cit, OA, SCoA, α-KG, and Iso were ~1.5–10 times higher than the control ones (mean values), and the levels of Fum, Pyr, Lac, and α-KGM were ~1.5–3 times lower (mean values). Additionally, according to previously published data, the content of ACoA in the same groups of rats was also significantly lower than the control values [13].

Acute hyperammonemia is characterized by a decrease in the level of Pyr and an increase in the level of Lac. In the liver tissues of TAA-induced rats of the post-rehabilitation period, the level of both metabolites was below the control values. Thus, instead of a decrease in the Pyr/Lac ratio, which is usually observed during periods of acute HE, here, on the contrary, it increased (Figure 4). This was especially noticeable in the group of rats with the highest dose of TAA, where the difference with the control group was ~2–8-fold. Moreover, the trends in the distribution of Pyr and α-KGM in the liver of rats, as well as in blood plasma, coincided, while the trends in the distribution of α-KG and Lac were multidirectional. The sharp increase in the Pyr/Lac ratio in the groups of TAA-induced rats that received high doses of TAA was due to a sharp decrease in the Lac concentration in these groups relative to control values (Figure 3 and Figure 4).

According to Lac distribution, it could be assumed that in the group of TAA-induced rats of the post-rehabilitation period, some processes were going on that were reverse to those that took place during the development of acute HE. These processes were all the more pronounced the larger the dose of toxin previously received by these animals.

It should be noted that under the same conditions, a marked decrease in the level of ACoA occurs [13]. ACoA is an intermediate product of Pyr formation and a source of TCA replenishment through Cit. In this study, we found that there was (in mean values) an increase in Iso, Cit, α-KG, and, to some extent, SCoA. It is noteworthy that in the liver of TAA-induced rats, an inverse relationship was observed in the content of α-KG and α-KGM, namely, with an increase in the level of α-KG, the level of α-KGM decreased, in contrast to the data obtained for blood plasma (Figure 1 and Figure 3). Accordingly, the calculated ratios of α-KG/α-KGM in the liver of TAA-induced rats differed 3-fold (200 mg/kg TAA), 4-fold (400 mg/kg TAA), and 28-fold (600 mg/kg TAA) relative to the control (Figure 4). In general, the change in this ratio between groups was even more pronounced than that in the Pyr/Lac ratios (maximum up to ~8-fold). However, the trends in both biomarker ratios were opposite to those found in plasma.

In general, α-KGM is mentioned in the context of a toxicity as a biomarker [15]. Therefore, a decrease in its concentration may possibly reflect an ongoing process of detoxification in the liver, and an increase in the level of α-KG may possibly reflect an intensification of recovery processes in liver tissue.

The nature of the distribution of the main metabolites in the kidney was somewhat different from that in the liver, which indicates differences in the processes involved in the recovery of these organs. At the same time, the nature of the distribution of metabolites in the kidneys of rats was in good agreement with the pattern of their distribution in the blood plasma, which may be a consequence of the direction of the work of the kidneys, which pass through a large volume of blood every day.

The levels of such TCA metabolites as ACoA [13], Mal, Lac, Fum, Suc, and Iso in the kidneys of TAA-induced rats were ~1.5–5 times higher (mean values) compared to the those of control group, while OA, Pyr, SCoA, α-KG, and α-KGM were ~1.5–4 times lower. Interestingly, in the liver of TAA-induced rats, the levels of SCoA and α-KG increased relative to those of the control, while in the kidneys, they decreased (Figure 3 and Figure 5). The levels of such metabolites as Suc, Lac, and ACoA [13] in the liver of TAA-induced rats decreased, while in the kidneys of the same groups of rats, on the contrary, they increased (Figure 3 and Figure 5). Different mechanisms involved in the liver and kidney restoration are likely to work for normalizing the balance of TCA after acute HE, which may be due to residual inhibition of specific groups of enzymes that prevail in the tissues of certain organs.

In this context, a ~2-fold decrease in the Pyr/Lac ratio in the kidneys of TAA-induced rats attracted attention, whereas an ~8-fold increase was noted in the liver of rats (Figure 4 and Figure 6). This circumstance was due to the persistence of elevated levels of Lac in the kidneys of TAA-induced rats relative to control values. Thus, it should be noted that according to the cumulative change in the amount of Pyr and Lac in the kidney tissues of TAA-induced rats, unlike the liver tissues, knock-on effects of acute HE remained even 6 days after the intoxication (Figure 3 and Figure 5). It is noteworthy that the increase in Lac concentration was also accompanied by an increase in the concentration of ACoA [13], which may be due to PDH inhibition.

In general, in the kidneys of TAA-induced rats, there was a decrease in the levels of both α-KG and α-KGM and, unlike liver tissues, this decrease did not occur in different directions, but synchronously. Obviously, in this regard, in the tissues of the kidneys of TAA-induced rats, only a ~2-fold increase in the ratio of α-KG/α-KGM was observed, and not ~28-fold, as in the liver (Figure 4 and Figure 6). 

At the same time, the uneven dose-dependent distribution of the α-KG/α-KGM ratio in the tissues of the kidneys attracts attention, in which the group of rats that received TAA at a dose of 400 mg/kg had a higher ratio relative to that of groups with lower and higher (200 and 600 mg/kg) doses of TAA. Individual indicators of rats revealed an animal with a different metabolic picture. Thus, the level of α-KG in the kidneys of this rat was significantly higher, and the level of α-KGM was significantly lower than the neighboring indicators. Since the balance of α-KG and α-KGM in the tissues of the organ is related, this circumstance reflects the individual metabolic pattern of the animal. Characteristically, a similar distribution of α-KG/α-KGM in the group of rats treated with TAA at a dose of 400 mg/kg was noted in the blood plasma constantly circulating through the kidneys (so-called interorgan communication) (see Figure 1, Figure 2, Figure 5 and Figure 6).

The concentrations (mean values) of metabolites such as SCoA, Pyr, α-KG, Cit, and Fum in the brain tissues of TAA-induced rats increased (Figure 7), while some others, namely OA, α-KGM, and ACoA [13] decreased; however, Lac and Mal changed slightly. Against the background of a slight increase in the concentration of Pyr in the brain of TAA-induced rats and a slight change in Lac parameters, the Pyr/Lac ratio increased slightly (Figure 8). In contrast to the ~8-fold increase in this ratio in the liver of TAA-induced rats with the maximal dose of TAA, the increase in this indicator in the brain tissues of rats was small, ~10–20%.

The concentration of α-KG in the brain tissues of rats increased in accordance with the increase in the dose of administrated TAA, and the level of α-KGM decreased in the opposite way. The noted pattern of distribution of these ketoacids in the brain tissue of experimental animals was close to that in the liver of rats. However, instead of a ~28-fold increase in the ratio of α-KG/α-KGM in the liver of rats that received the maximal dose of TAA, the increase in this ratio in the brain tissues of the same rats was ~5-fold (Figure 3 and Figure 7). This is not surprising due to the greater protection of the brain from exposure to toxic substances. Here, a ~2-fold increase in the level of α-KG was accompanied by a ~2-fold decrease in the level of α-KGM. Perhaps this was due to the different activity of the enzyme ω-amidase, the amount of which in the liver of rats is much higher than in the brain tissues (not published data, manuscript in preparation).

Despite the long recovery period after HE and the objective physiological improvement in the condition of the animals, after a thorough statistical analysis (see Supplement S1), a statistically significant difference was noted between the control and TAA-induced groups of rats in the concentrations of TCA and associated metabolites in the following tissues: SCoA in the plasma; Suc, α-KG, and α-KGM in the liver; SCoA and Iso in the kidney. Our findings reflect that: firstly, the results showed a trend aimed at stabilizing the mechanisms of energy metabolism in the body as a whole, and secondly, in such vital organs as the liver and kidneys, there was a residual imbalance of energy-important metabolites of the Krebs cycle.

Thus, the obtained results make it possible to use these data both for prognostic purposes and in the selection of potential therapeutic agents.

## 4. Materials and Methods

### 4.1. Reagents 

TAA and reagents for the preparation of TCA standards of the highest quality were obtained from Sigma–Aldrich (St. Louis, MO, USA). The α-KGM standard was synthesized in the laboratory, the purity of the preparation was confirmed by mass spectrometric analysis. Potassium phosphate dibasic, ultrapure, powder reagent, and perchloric acid, 69–72%, were obtained from J.T. Baker (Philipsburg, NJ, USA). Ultrapure water was obtained using a Milli–Q Gradient A10 system (Millipore, Bedford, MA, USA). All chemicals were of HPLC grade and were used without further purification. For mobile phase filtration and degassing, nylon membrane filters of 47 mm, pore size 0.2 μm, obtained from Pall Life Science (Port Washington, NY, USA) were used.

### 4.2. Rat Model of Liver Failure 

Eighteen 4-month-old Wistar rats, females, 130–140 g of weight were used in the experiment. For 2 weeks prior to the start of the experiment, the rats were quarantined with a 12 h light cycle and received normal food and free access to drinking water.

TAA for injection was prepared in saline and sterilized by passing through a Millipore filter with a pore size of 0.2 μm. The control group of rats (n = 3) received a single intraperitoneal injection of sterile saline. The remaining rats received TAA in doses of 200 (n = 3), 400 (n = 6), and 600 (n = 6; 2 animals died during experiment) mg/kg.

Rat blood plasma, liver, kidney, and brain samples collected on the sixth day after the TAA injection were immediately frozen in liquid nitrogen and stored at −80 °C until HPLC analysis. It should be noted here that due to the lack of information on the balance of metabolites in the organs of rats in their post-rehabilitation period after TAA intoxication, it was decided to conduct a preliminary study on the whole organ of rats, and not on region-specific areas. All animal work was carried out in accord with the institutional guidelines for animal use. Animal Protocol № 22/1 approved by the Supervisory Board of the Bach Institute of Biochemistry and by the Bioethics Committee of the Biotechnology Research Center of the Russian Academy of Sciences, Moscow.

### 4.3. Preparation of Samples for HPLC Analysis 

The method of analysis of α-KGM and TCA metabolites published previously was used [16,18]. The HPLC configuration consisted of a Waters 2489 UV/VIS detector, a Waters 1525 binary pump, a Waters 2707 autosampler with a cooled platform (4 °C), a C18 analytical column, YMC, Triart, 250 × 3.0 mm, 3 μm with a Phenomenex Security guard column (C18, 4 × 2 mm). The columns were kept at room temperature, the injection volume was 15 μL at an isocratic flow rate of the mobile phase (20 mM KH_2_PO_4_, pH 2.9) of 0.45 mL/min. Metabolites were determined at a wavelength of 210 nm. Sample analysis was controlled using Breeze2 software (Waters Corporation) installed on a Dell computer.

Identification and quantification of individual metabolites was performed against appropriate standards of known concentration. The levels of their content in blood plasma were calculated (µM), and in tissue samples they were recalculated per milligram of wet weight.

### 4.4. Preparation of Tissue Samples for Analysis 

To 10–20 mg of tissue, 400 µL of chilled 10% PCA was added, briefly vortexed until the sample was immersed in the solution. Brain tissues were homogenized for 6 s at an amplitude of 15%. The tissues of the liver and kidneys were homogenized in two sets (2 × 6 sec) at an amplitude of 20%. For better extraction of metabolites, the homogenate was kept on snow for 10 min, shaking occasionally, and then centrifuged at 14,000× *g* (4 °C) for 20 min. The resulting supernatant was transferred to a clean Eppendorf and centrifuged again. Up to 100 µL of the resulting extract was transferred into a vial for direct injection into the HPLC system. 

### 4.5. Preparation of Plasma Samples for Analysis 

To 50 μL of plasma, 250 μL of an ice-cold solution of 10% PCA was added, vortexed for 1 min, and centrifuged as described above.

### 4.6. Statistics 

Data are presented as mean ± standard deviation (AVG ± STDEV) with calculated *p*-values by Tukey’s test. The choice of a statistical method of analysis was based on such prerequisites as a small and unequal sample size in groups of animals and high individual variability of metabolites. The analysis was focused on identifying a possible relationship between the quantitative characteristics of individual metabolites (for example, the concentration of α-KG and α-KGM in samples) and such qualitative parameters as the diversity of biological samples (liver, kidney, brain, and blood plasma) and the dose of administered TAA (200, 400, and 600 mg/kg). The project involved four groups of animals, 3–6 rats in each. Up to 40 measurements were performed for each animal (9–11 metabolites in four types of biological samples). A part of the metabolites was not included in the statistical processing due to implicit trends depending on the doses of TAA used.

Forty measurements were performed for each rat (n = 16, 9–11 TCA metabolites in four types of samples). The approach was similar to that published previously [13]. Briefly, the relationship between the TCA metabolite concentrations and their quantitative features in different types of samples with different doses of TAA (0, 200, 400, and 600 mg/kg) was studied (for details, please see Supplement S1). There were 120 independent hypotheses formulated on the absence of a difference between the concentrations of TCA metabolites and their derivatives in the control and experimental groups (9–11 TCA metabolites and their derivatives, control and 3 groups of animals treated with TAA, 4 types of tissues) and 120 independent hypotheses on the absence of differences between experimental groups of animals (9–11 TCA metabolites and their derivatives, 3 pairs of groups, 4 tissue types). Generally, 240 initial hypotheses were involved in the analysis. Multivariate analysis of variance (MANOVA) and Tukey’s test were used to test the hypotheses. For multiple hypothesis testing, the Benjamini–Hochberg procedure was used. After the checks, a two-factor analysis of variance was performed for pairs of dependent variables (TCA metabolite concentrations from one type of tissue).

## 5. Conclusions

Thus, summarizing what was mentioned above, we can conclude that:

In a six-day period from the moment of intoxication of rats with various doses of TAA, the levels of the main metabolites of TCA and a number of concomitant metabolites in the blood plasma, tissues of the liver, kidneys, and brains of rats retained traces of intoxication.

The levels and distribution of the main metabolites in the blood plasma and organs of the animals in the post-rehabilitation period indicate the presence of various mechanisms for their recovery.

The pattern of distribution of levels of TCA metabolites in rat tissues after recovery from TAA exposure indicated marked affinity in vital organs and/or media such as rat liver and brain, and rat kidney and blood plasma.

Identified disorders of metabolic pathways in the plasma and organs of animals of the post-recovery period can be used as potential markers for monitoring the recovery process after acute HE, as well as for assessing the effectiveness of potential therapeutic agents.

## Data Availability

Additional data may be available upon personal request.

## Supplementary Materials

The following supporting information can be downloaded at: https://www.mdpi.com/article/10.3390/ijms24021384/s1.
