# Supplementary material for: Tricarboxylic Acid Metabolite Imbalance in Rats with Acute Thioacetamide-Induced Hepatic Encephalopathy Indicates Incomplete Recovery
Source: Int J Mol Sci. 2023 Jan 10;24(2):1384. doi: 10.3390/ijms24021384 (PMC9861856; doi:10.3390/ijms24021384)
Supplement: Supplementary file 1 [file ijms-24-01384-s001.zip › ijms-2062184-supplementary.pdf]

Supplement S1

Statistical analysis was performed using the R language.

Initial data:

- 16 rats;
- Divided into 4 groups: 3, 3, 6, 4 individuals in groups;
- For each individual, the results of 40 measurements were analyzed. For Plasma and Liver samples, data were obtained on the concentration of 11 TCA metabolites and their derivatives, namely: succinyl coenzyme A (SCoA), oxaloacetate (OA), pyruvate (Pyr), α-ketoglutaramate (α-KGM), malate (Mal), isocitrate (Iso), α-ketoglutarate (α-KG), lactate (Lac), citrate (Cit), fumarate (Fum), succinate (Suc). For the other two tissues, the concentration values of 10 metabolites for Kidney (SCoA, OA, Pyr, α-KGM, Mal, Iso, α-KG, Lac, Fum, and Suc) and 9 metabolites for Brain (SCoA, OA, Pyr, α- KGM, Mal, α-KG, Lac, Cit and Fum) were obtained.

The approach to identifying possible patterns used in this study was similar to that used by us previously (Ref. 13 in this paper; https://doi.org/10.3390/ijms21238918), specifically, the relationship between the concentration values of 9-11 TCA metabolites and their derivatives (quantitative traits) in different types of tissue (plasma, liver, kidney, brain) and groups of experimental animals with different administered doses of TAA (0 - control group, 200, 400 and 600 mg/kg (qualitative factors)) was studied.

There were 120 independent hypotheses formulated on the absence of a difference between the concentrations of TCA metabolites and their derivatives in the control and experimental groups (9-11 TCA metabolites and their derivatives, control and 3 groups of animals treated with TAA, 4 types of tissues) and 120 independent hypotheses on the absence of differences between experimental groups of animals (9-11 TCA metabolites and their derivatives, 3 pairs of groups, 4 tissue types). In total, the number of initial hypotheses was 240.

Multivariate analysis of variance (MANOVA) and Tukey's test were used to test the hypotheses. For multiple hypothesis testing, the Benjamini-Hochberg procedure was used.

To use multivariate analysis of variance, it is necessary that the data meet a number of criteria.

First, for the correct application of multivariate analysis of variance, the number of observations in each group must exceed the number of dependent variables (in our case, the number of TCA metabolites and their derivatives). Since in our study there are 3 observations in each group, multivariate analysis of variance can be performed for two variables. Therefore, the analysis was carried out for different pairs of dependent variables.

Second, the data within the groups must be normally distributed. To check the normality of distributions, the Shapiro-Wilk test was used (a total of 40 tests for each dependent variable (TCA metabolite concentration or metabolite derivative) in each group, R function - shapiro.test()). The null hypothesis of normal distribution was rejected (at a 5% significance level) for OA in brain and kidney tissues, Lac in kidney tissue in the control group, for Lac in plasma in the TAA 200 mg/kg dose group, for KGM, Fum in tissue kidneys, Pyr, Lac - in the liver tissue in the group with a dose of TAA 400 mg/kg. Measurements for these TCA metabolites and derived metabolites in the respective organs were excluded from consideration. These tests were performed on independent datasets, so no multiple hypothesis testing adjustment was required. Subsequent analysis was performed for all possible pairs of dependent variables (concentrations of TCA metabolites and their derivatives).

In addition to checking the normality of data distributions within groups, it is necessary to check for normality of the joint distribution of the variables under study, evaluate the degree of correlation between variables to test for multicollinearity, check the data for the lack of homogeneity of the covariance matrix, and check the equality of variances for dependent variables in different groups. 703 normality tests of the two-dimensional distribution of concentrations of TCA metabolites and their derivatives in different organs (using the mshapiro_test() function), 703 correlation estimates (using the cor_test() function), 703 tests for the homogeneity of covariance matrices (using the box_m() function) were carried out and 40 equal variance tests (using the bartlett.test() function). For multiple hypothesis testing, the Benjamini-Hochberg procedure was used. Some pairs of variables did not pass the test, their study using multivariate analysis of variance was not conducted.

After checks, a two-way analysis of variance was performed for pairs of dependent variables (concentrations of TCA metabolites and their derivatives from the same type of tissue). There was a significant difference in the concentrations of TCA metabolites or their derivatives for different tissues and the concentration of TAA (shown in Table S1).

The same analysis was carried out for KG/KGM and Pyr/Lac ratios. The tests for normality, multicollinearity and homoscedasticity for 8 additional dependent variables were used. All the variables passed the tests. The ANOVA test showed significant differences in mean values of ratios KG/KGM for liver tissue (at 5% sinificance level), and Pyr/Lac for kidney tissue and KG/KGM for brain tissue and plasma (at 10% significance level). Results of Tukey tests for ratios are also presented in Table S1.

Table S1. Pairwise results comparison*.

TCA	Tissue	***p*-values (Tukey test)**					
		**200 to 0**	**400 to 0**	**600 to 0**	**400 to 200**	**600 to 200**	**600 to 400**
SCoA	Kidney	0.003	0.002	0.001	0.996	0.948	0.819
Iso	Kidney	0.568	0.022	0.125	0.268	0.746	0.799
KGM	Liver	0.096	0.007	0.001	0.700	0.119	0.362
KG	Liver	0.228	0.130	0.002	0.995	0.052	0.053
Suc	Liver	0.002	0.000	0.000	0.956	0.787	0.944
SCoA	Plasma	0.075	0.031	0.416	1.000	0.565	0.379
KG/KGM	Liver	0.654	0.210	0.002	0.821	0.011	0.029
Pyr/Lac	Kidney	0.095	0.065	0.073	0.998	1.000	0.998
KG/KGM	Brain	0.999	0.485	0.079	0.810	0.237	0.320
KG/KGM	Plasma	0.411	1.000	0.166	0.314	0.950	0.092

* Experimental groups were compared to control "0", and among groups TAA-treated with 200, 400, and 600 mg/kg.

Colour	Significance
(orange)	$p < 0.005$ - ***
(blue)	$p < 0.05$ - **
(green)	$p < 0.1$ - *

To determine between which groups of rats (with different doses of TAA) there is a significant difference in these metabolites, the Tukey test was used, which involves pairwise comparison of group averages with the overall average. Tukey's test was implemented using the TukeyHSD() function for 6 independent variables (no test was performed for Suc in plasma due to lack of data).

The results obtained (Figures S1–S6) indicate a significant (at 5% level) difference between the concentrations in the control group and the remaining groups of SCoA in kidney tissue and α-KG

in liver tissue. Significantly different concentrations of Iso in kidney tissue, SCoA in plasma and α-KGM in liver tissue in the control group and in the group with a dose of TAA 400 mg/kg. There is also a significant difference between the concentrations of α-KG and α-KGM in the liver tissue in the control group and in the group with a dose of TAA 600 mg/kg.

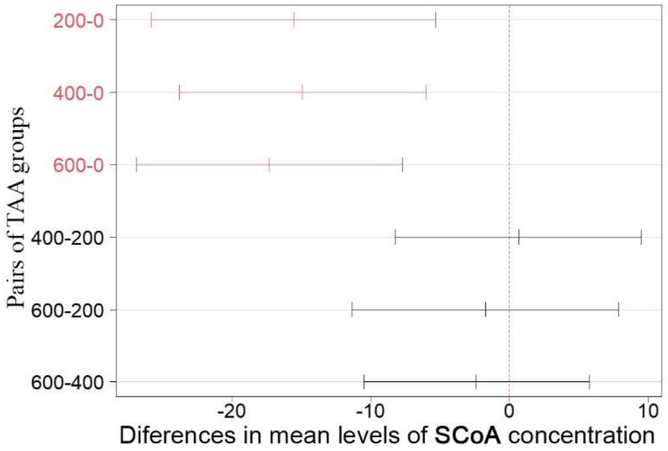

Figure S1. Differences in mean levels of SCoA concentrations obtained from kidney tissues for the different pairs of TAA groups and 95% family-wise confidence limits.

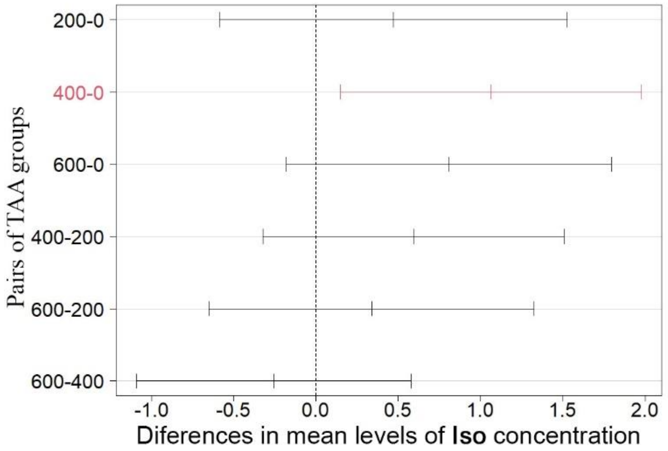

Figure S2. Differences in mean levels of Iso concentrations obtained from kidney tissues for the different pairs of TAA groups and 95% family-wise confidence limits.

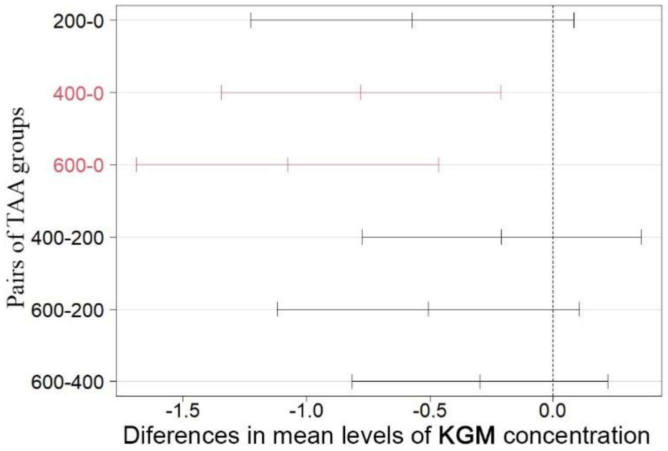

Figure S3. Differences in mean levels of α-KGM concentrations obtained from liver tissues for the different pairs of TAA groups and 95% family-wise confidence limits.

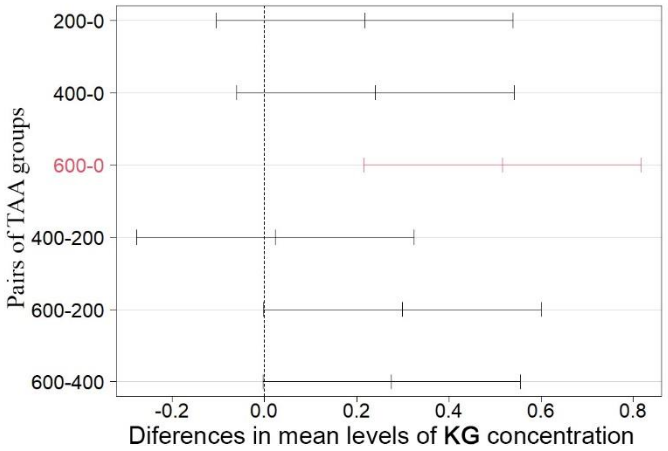

Figure S4. Differences in mean levels of α-KG concentrations obtained from liver tissues for the different pairs of TAA groups and 95% family-wise confidence limits.

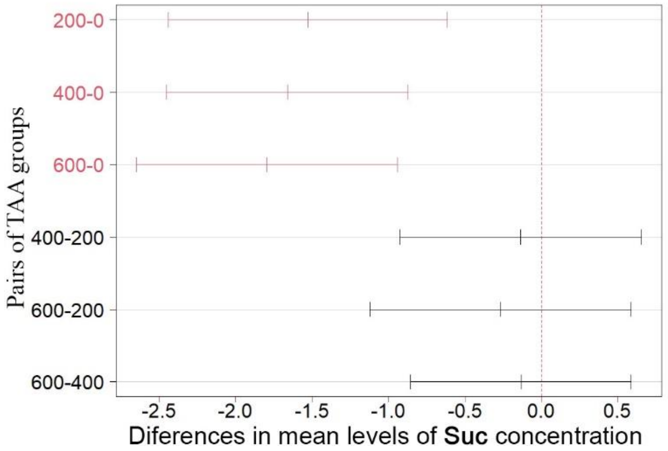

Figure S5. Differences in mean levels of Suc concentrations obtained from liver tissues for the different pairs of TAA groups and 95% family-wise confidence limits.

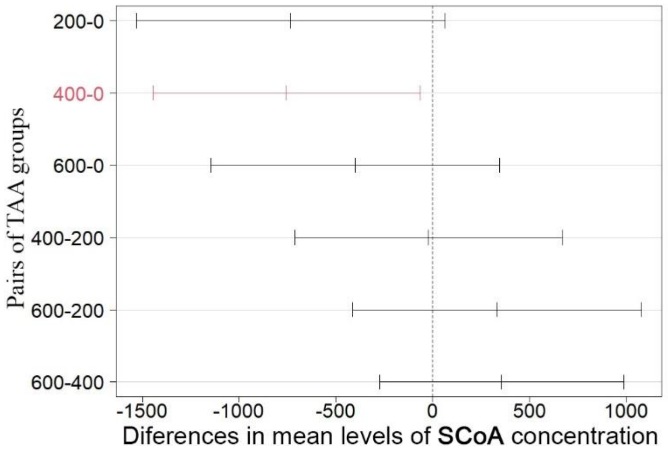

Figure S6. Differences in mean levels of SCoA concentrations obtained from plasma samples for the different pairs of TAA groups and 95% family-wise confidence limits.

Preliminary data analysis in Figures S7–S11 is presented as median, 25% and 75% as quartiles and outliers.

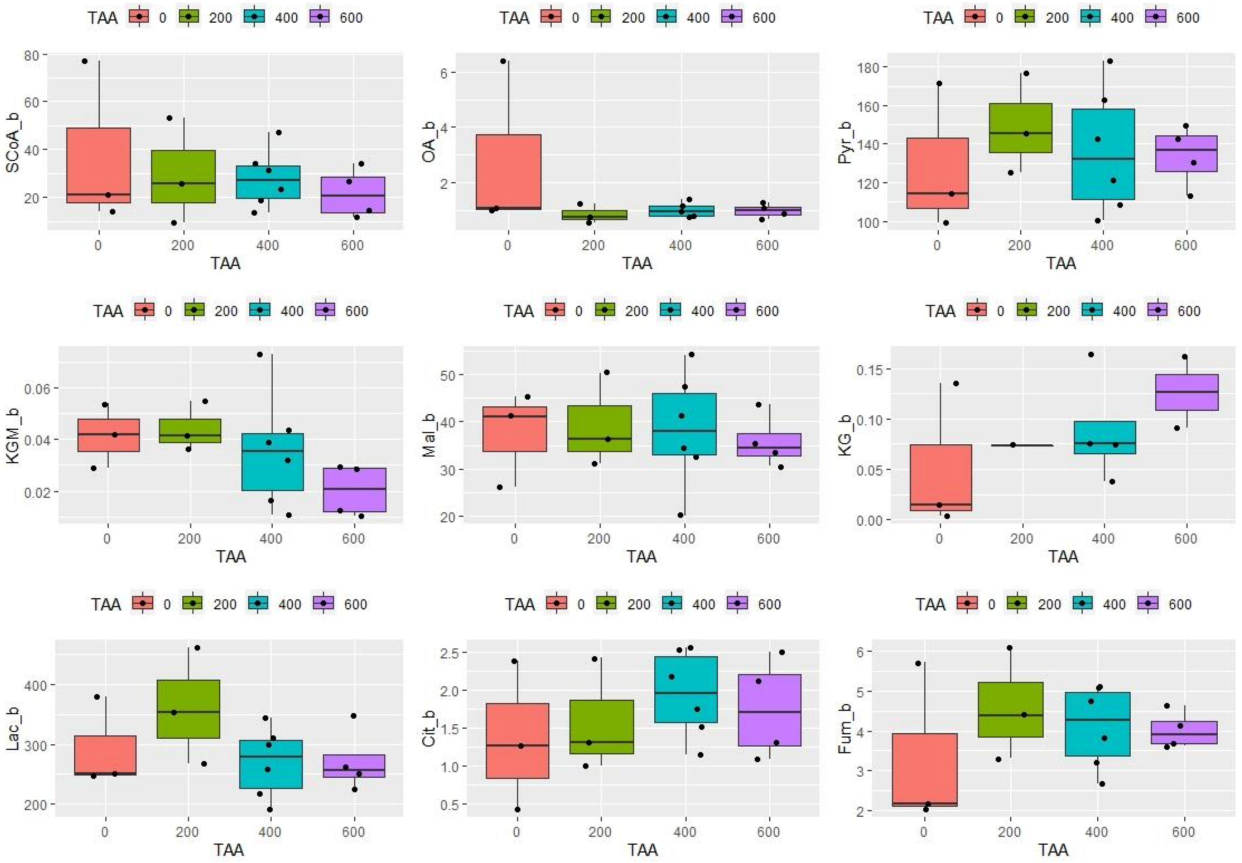

Figure S7. Boxplots (median, 25% and 75% quartiles and outliers) of the TCA metabolites and their derivatives concentrations obtained from the brain tissue.

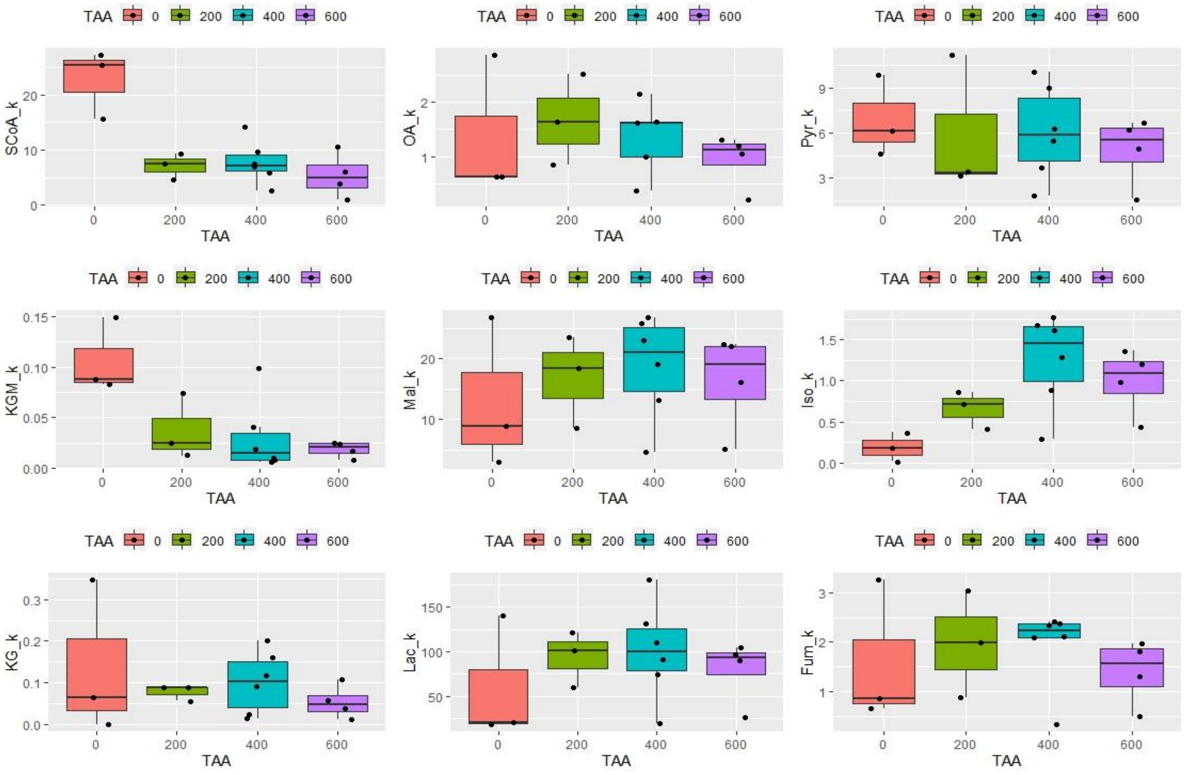

Figure S8. Boxplots (median, 25% and 75% quartiles and outliers) of the TCA metabolites and their derivatives concentrations obtained from the kidney tissue.

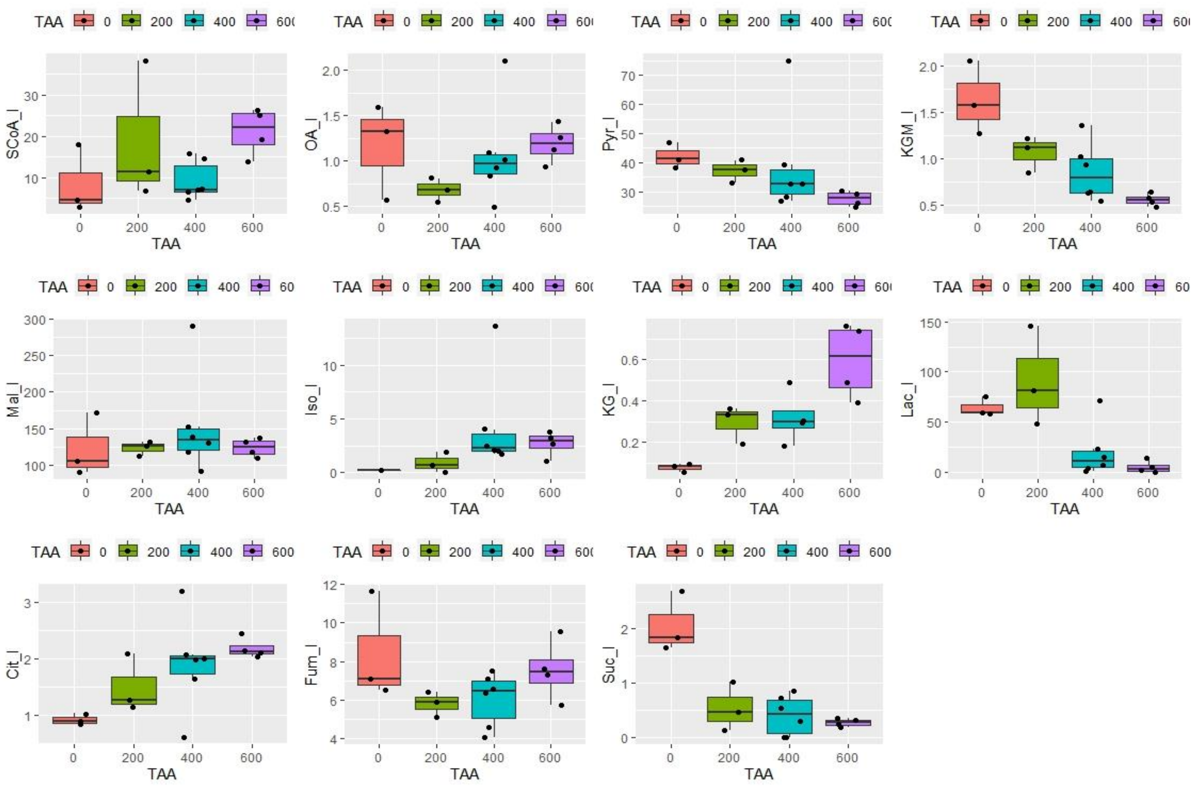

Figure S9. Boxplots (median, 25% and 75% quartiles and outliers) of the TCA metabolites and their derivatives concentrations obtained from the liver tissue.

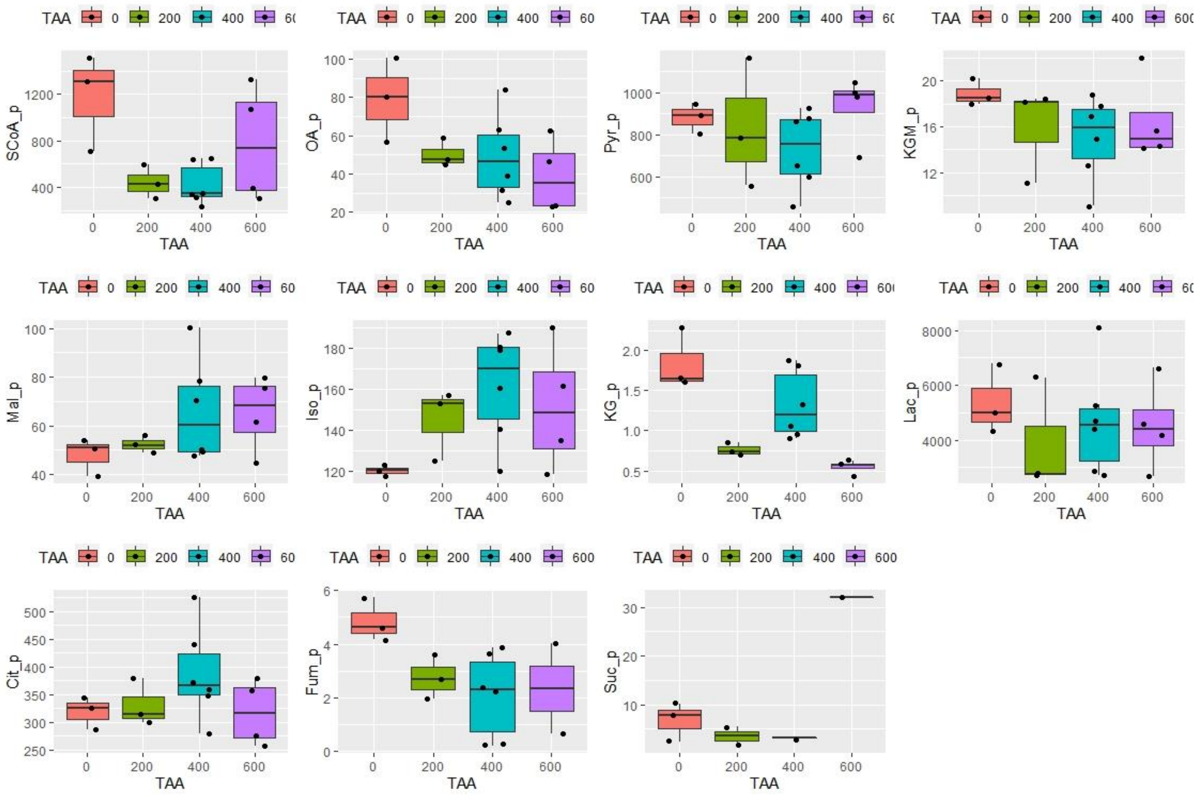

Figure S10. Boxplots (median, 25% and 75% quartiles and outliers) of the TCA metabolites and their derivatives concentrations obtained from the plasma samples.

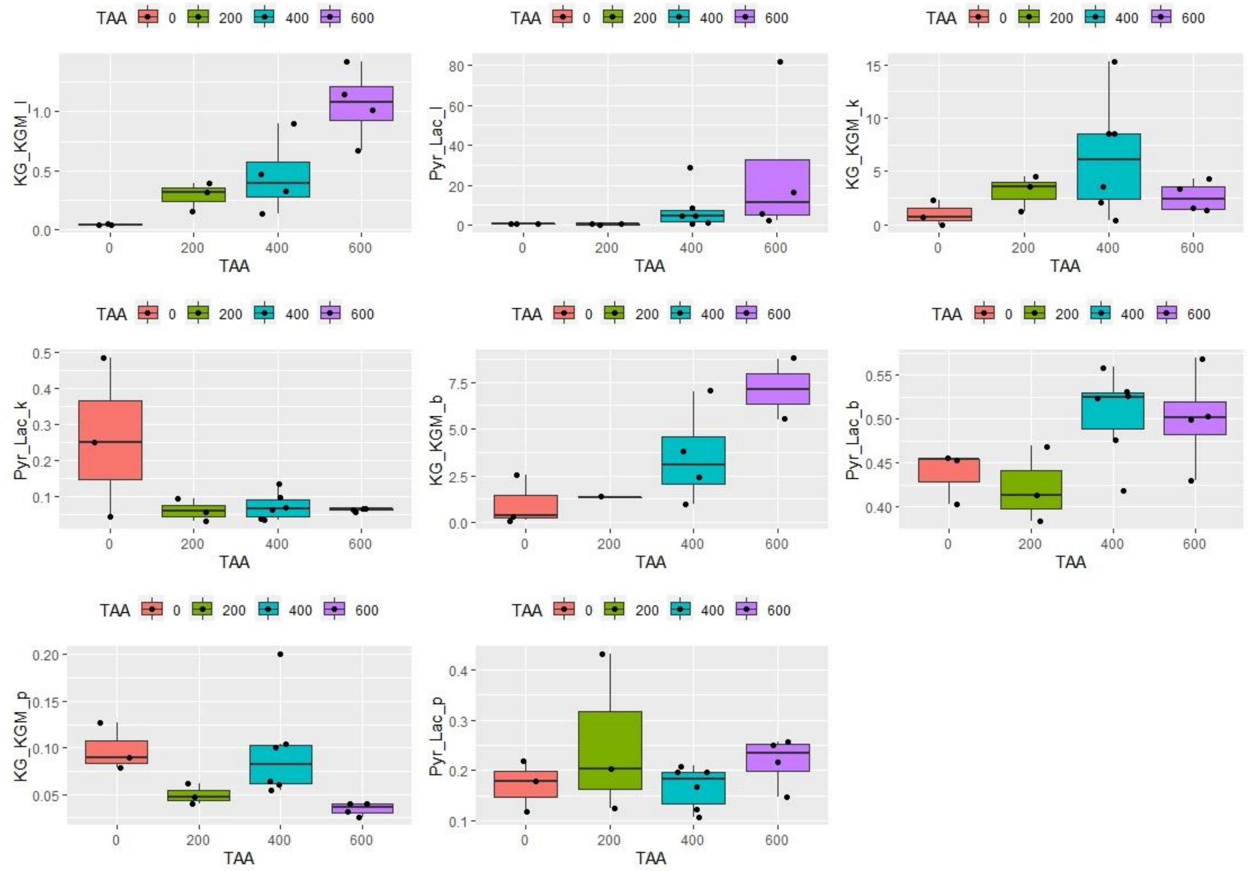

Figure S11. Boxplots (median, 25% and 75% quartiles and outliers) of the KG/KGM and Pyr/Lac ratios calculated for l – liver, k – kidney, b – brain tissues and p - plasma samples.
